# Innovation in the Processing of Native Round Fish: A Readjustment of the Processing Workflow for *Salmonella* spp. Control in a Fish Processing Plant in the State of Mato Grosso

**DOI:** 10.3390/ani15121679

**Published:** 2025-06-06

**Authors:** Jaqueline Oliveira Reis, Nathaly Barros Nunes, Yuri Duarte Porto, Adelino Cunha Neto, Sara Rodrigues de Souza, Washington da Guia Fonseca, Alexsandro da Silva Siqueira, Luciana Kimie Savay-da-Silva, Eduardo Eustáquio de Souza Figueiredo

**Affiliations:** 1 Faculty of Agronomy and Zootechnics, Federal University of Mato Grosso (UFMT), Cuiabá 78060-900, Mato Grosso, Brazil; jaqueline.reis@sou.ufmt.br (J.O.R.);; 2 Faculty of Nutrition, Federal University of Mato Grosso (UFMT), Cuiabá 78060-900, Mato Grosso, Brazil

**Keywords:** *Salmonella* spp., fish processing plant, chlorine, processing workflow, food safety

## Abstract

*Salmonella* spp. is a pathogen detected in fish, although it is not part of its microbiota; the production and processing environment is the main source of contamination. Brazilian legislation recommends 5 ppm of free residual chlorine for fish washing, but *Salmonella* can still be present. The objective of this study was to evaluate flaws in the processing flowchart and propose adjustments to reduce *Salmonella* spp. on the fish surface. Ninety samples were analyzed in a fish processing plant in Mato Grosso, divided into three treatments: (1) conventional processing, (2) modified flowchart, and (3) modified flowchart adapted to the specific plant conditions. Treatment 2 completely eliminated *Salmonella* spp., while treatment 3 reduced contamination to 3.3%, compared to 56.7% in conventional processing. The success of the modified treatment was only possible due to the main changes implemented in the process, which included the separation of dirty areas (responsible for gill and scale removal) and clean areas (designated for the careful removal of viscera without rupture and for filleting). No statistical difference was found between treatments 2 and 3 (*p* = 1, CI 0.00000–39.00055), suggesting that the adjusted flowchart can be implemented on a large scale to ensure food safety (OR = ∞, CI = [7.655, ∞], *p* < 0.001). This study highlights the effectiveness of the adjusted flowchart in reducing *Salmonella* spp. contamination in fish, with treatment 2 resulting in a complete absence of contamination. Treatment 3 maintained low contamination levels, demonstrating practical applicability in meatpacking plants.

## Simple Summary

The presence of *Salmonella* spp. in fish poses a risk to public health, even though this bacterium is not part of the natural microbiota of fish. The main source of contamination is the production and processing environment. In Brazil, washing with chlorinated water at 5 ppm is required, but this measure is not always effective. This study was conducted in a fish processing plant in the state of Mato Grosso to identify failures in the processing flowchart and propose improvements. Three treatments were tested: the conventional process, a modified flowchart, and a modified flowchart adapted to the plant’s structure. The modified treatment completely eliminated *Salmonella* spp., while the adapted version reduced contamination from 56.7% to only 3.3%. The main change was the physical separation between dirty areas (gill and scale removal) and clean areas (evisceration and filleting), reducing cross-contamination. The results show that simple adjustments in the process can significantly increase the safety of the final product. Applying these measures on an industrial scale may contribute to the production of fish that is safer from a hygienic and sanitary standpoint for the consumer.

## 1. Introduction

The fisheries and aquaculture sectors have been gaining increasing recognition for their strategic importance in promoting global food and nutrition security. Projections indicate that, by 2032, these sectors are expected to experience significant growth in production, consumption, and trade. Total aquatic animal production is expected to reach 205 million tons by 2032, with the primary increase coming from aquaculture, contributing 101 million tons in 2030 [1]. According to the Brazilian Fish Farming Association Peixe BR [2], the production chain of farmed fish in Brazil ranks seventh in the world for freshwater fish production. Regarding native fish production, Brazil produced 258,705 tons in 2024. Among the five Brazilian states considered the largest producers of native round fish from the Amazon, Mato Grosso was the only one to show an increase in production, moving from third place in 2022 to second place in 2023, reaching a production of 44,520 thousand tons in 2024.

Despite its high production, consumption, and nutritional importance, fish is considered an animal-based food particularly vulnerable to microbiological contamination [3], posing a significant risk to food safety. Among the main pathogens associated with this type of product, *Salmonella* spp. stands out, with its presence linked both to the natural habitat conditions of fish and to inadequate handling and processing practices. *Salmonella* contamination in fish represents a public health threat, requiring effective control measures throughout the production chain [4].

Pathogenic microorganisms such as *Salmonella* spp. can be introduced into fish and their final products during both the farming and processing stages [5]. While *Salmonella* spp. is not part of the fish microbiota, it can be introduced through the farming environment, such as through contaminated water, sediments, and other factors [6]. Fish contamination in processing plants can occur at all stages of processing, including transportation, washing with hyperchlorinated water, evisceration, scaling, and filleting, as well as through contact with contaminated water, cutting boards, knives, trays, and plastic boxes. Parts such as scales, gills, and viscera can potentially be contaminated and can transfer microorganisms to the fish during processing, increasing the risk of contamination [7]. Additionally, there are other factors that may promote the persistence of this microorganism in fish, which are currently being investigated by our research group, such as the interaction of organic matter with chlorine effectiveness and the exposure time of *Salmonella* to chlorine [8,9]. Therefore, protective measures against foodborne pathogens should be implemented, as well as ensuring proper sanitary conditions from processing to commercialization of the fish.

To ensure the quality of final products, the Ministry of Agriculture and Livestock (MAPA) mandates the use of a surface wash with 5 ppm of free residual chlorine. This wash is the only measure that has been proposed to prevent the persistence of pathogenic microorganisms on fish surfaces [10]. Despite the mandatory use of chlorine in fish washing, *Salmonella* serotypes have been detected in water, fish (such as tambaqui and pintado), and production farms in the state of Mato Grosso [11], as well as in processing plants (Fernandes et al., 2018) [7]. What is most concerning is that the bacteria have also been found in a study conducted by our research group in products ready for commercialization in the state of Mato Grosso, such as eviscerated and frozen fish, frozen tambaqui belly, and frozen pintado fillets [12]. These fish certainly underwent the required hyperchlorinated water washing process, yet the bacteria remained present. This highlights the serious public health risk, as these products, intended for direct consumption, could lead to severe infections in consumers, underscoring the need for more effective control measures.

Given the risks associated with *Salmonella* spp. contamination in fish and the need to improve sanitary procedures during processing [13], this study aimed to test an alternative surface washing protocol and adapt an industrial-scale slaughter flowchart for the control of *Salmonella* spp. in native round fish.

## 2. Materials and Methods

### 2.1. Experimental Design

An experiment was conducted at a native round fish processing plant in Cuiabá, in the state of Mato Grosso. This medium-sized facility processes approximately 7 tons of fish per day. The fish species selected was a hybrid (*Colossoma macropomum* × *Piaractus brachypomus*), sourced directly from an excavated tank within the property, with an average weight of 976.1 ± 313.4 g (mean ± standard deviation; *n* = 90). The fish were harvested with fishing nets and slaughtered through thermal shock stunning for one hour. There was no need for artificial contamination, as the property was already experiencing contamination in the excavated tanks. Additionally, prior *Salmonella* analyses had confirmed the presence of the bacteria in both the tank and the fish.

### 2.2. Treatments

The treatments were divided into three flowcharts: the first simulated the fish processing performed by the establishment, while the other two represented modified flowcharts. Before being applied on an industrial scale, these flowcharts were tested on a laboratory scale. A total of 90 final product samples (whole carcasses) were analyzed, distributed among the three treatments.

#### 2.2.1. Treatment 1

**Figure 1:**
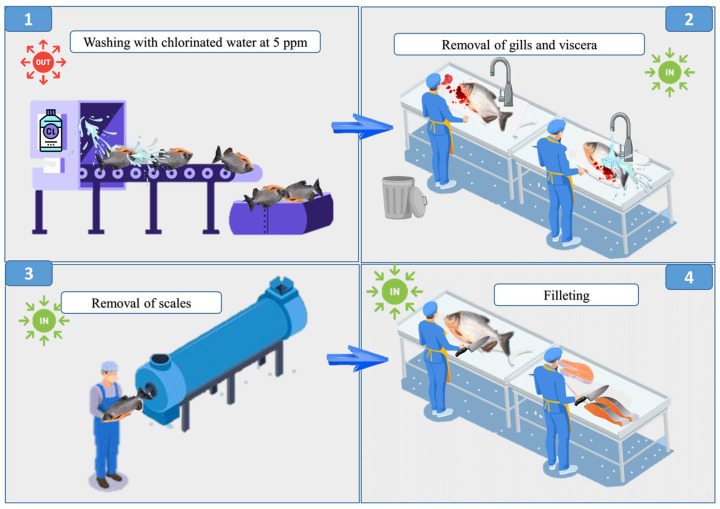
Workflow followed in the native round fish processing plant observed in this study. Captions: **1**: Surface washing with hyperchlorinated water at a concentration of 5 ppm of free residual chlorine before the fish enter the processing plant. **2**: After the fish enter the processing plant (clean area), the gills and viscera are removed, following a wash with tap water. **3**: Removal of fish scales in a rotating drum scaler. **4**: At this point, the carcasses are ready to proceed to the filleting stage. In: Within the clean processing area. Out: Outside the clean processing area.

Thirty samples were collected after undergoing the routine processing workflow of the facility, which is similar to the processing applied by the fish processing plant, as shown in Figure 1, below.

#### 2.2.2. Treatment 2

**Figure 2:**
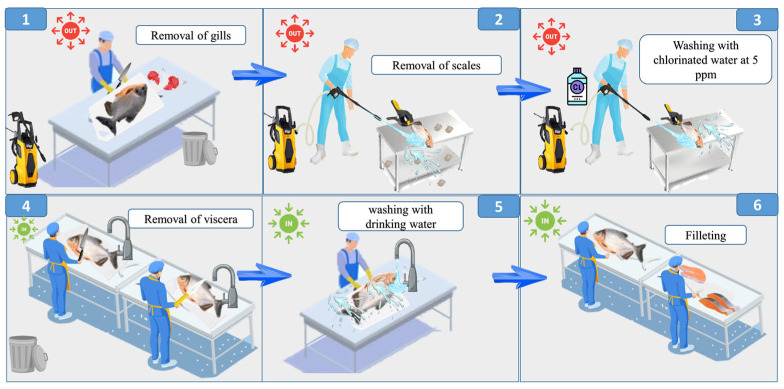
Workflow followed for the improved washing method in the native round fish processing plant. Captions: **1**: Removal of the gills, followed by washing with a low-pressure pump to remove blood residues. **2**: Removal of the scales using the pressure pump. **3**: Washing the fish with hyperchlorinated water at 5 ppm using the low-pressure pump. **4**: Opening the fish’s abdominal cavity, followed by the removal of the viscera. **5**: Washing with potable water. **6**: Filleting stage. In: Within the clean processing area. Out: Outside the clean processing area.

Thirty fish were collected after undergoing the improved processing workflow. The main changes implemented included the removal of the gills in an external area considered a dirty area, which occurred in the first stage. In the second stage, also in the external area, the scales were removed using a pressure pump. After this stage, the fish were washed with hyperchlorinated water at 5 ppm, still in the external environment, using the pump at minimum pressure, as shown in Figure 2. After this wash, the fish entered the processing plant for evisceration, which was performed by two workers: one to open the fish’s abdominal cavity and another to remove the viscera, ensuring that the intestinal contents did not come into contact with the carcass. Following this, the fish underwent another wash with potable water, both internally and externally, and were ready to proceed to the filleting stage. If the viscera ruptured, the fish were discarded, the workbench was washed with hyperchlorinated water at 25 ppm (a concentration used exclusively for surfaces in contact with fish), following the recommendation of the FAO (2008) [14], the knives and cutting boards were replaced, and the handlers sanitized their hands and changed gloves.

#### 2.2.3. Treatment 3

**Figure 3:**
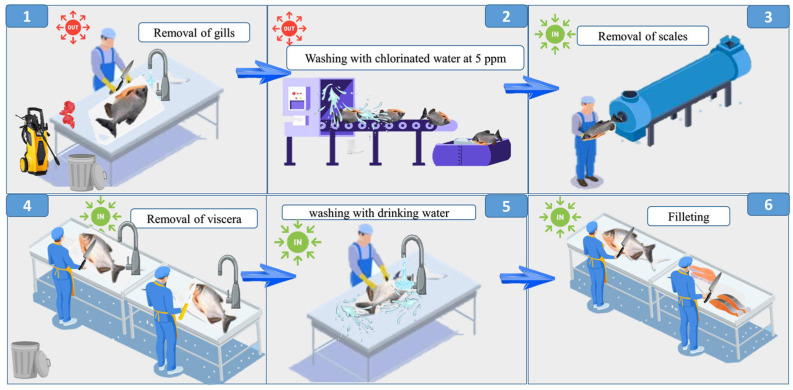
Processing workflow followed with the improved washing method, adapted to the native round fish facility. Caption: **1**: Removal of the gills, followed by washing with potable water to remove blood residues. **2**: Washing with hyperchlorinated water at 5 ppm using the facility’s equipment. **3**: Removal of the scales using the facility’s rotating drum scaler. **4**: Opening the fish’s abdominal cavity, followed by the removal of the viscera. **5**: Washing with potable water. **6**: Filleting stage. In: Within the clean processing area. Out: Outside the clean processing area.

An approach combining the modified treatment with the specific conditions of the facility was used, as shown in Figure 3. In this treatment, the removal of the gills was carried out in an external area, outside the processing zone. The fish were then washed with potable water using a low-pressure water pump. Only after this step were the fish subjected to washing with hyperchlorinated water at 5 ppm, using the facility’s equipment (a washing conveyor with a spraying system), and then the fish were directed to the processing area inside the plant. In the processing plant, the next step was the removal of the scales, performed with the facility’s scaling equipment. After scaling, the fish proceeded to the evisceration stage, carried out by two workers: one responsible for opening the abdominal cavity and the other for removing the viscera, ensuring that they did not rupture during the process. In cases of viscera rupture, the fish were discarded, and the workbench, materials, and utensils were sanitized with hyperchlorinated water at 25 ppm, following the recommendation of the FAO (2008) [14] for surfaces in direct contact with fish. Knives and cutting boards were replaced, and handlers sanitized their hands and changed gloves to maintain the hygiene of the environment.

### 2.3. Sample Collection

The washing of the eviscerated and chilled carcass (final product) was carried out according to the ISO 17604:2015 protocol [15]. According to the protocol, a sterile peptone saline solution was prepared, with a volume in the proportion of 1:4 relative to the weight of the fish (for example, for a 1000 g carcass, 250 mL of the solution was used). The fish was transferred to a sterile container, and 250 mL of peptone saline solution was added. The container was shaken manually, rotating the liquid inside to wash the entire internal and external surface of the fish for approximately 3 min. The resulting liquid was used for presence/absence tests to be applied in the subsequent stages of analysis.

### 2.4. Research on Salmonella spp.

The method used for the recovery and isolation of *Salmonella* spp. was essentially divided into four steps. In the first pre-enrichment step, 25 mL of the wash sample was inoculated into 225 mL of the non-selective liquid medium, buffered peptone water (BPW). These samples were incubated at a temperature of 37 ± 1 °C for 18 ± 24 h [16].

### 2.5. Enrichment in Selective Liquid Culture Medium

From the pre-enrichment broth, 0.1 mL aliquots were inoculated into Rappaport–Vassiliadis soya broth (RVS broth), and 1.0 mL aliquots were inoculated into Muller–Kauffmann tetrathionate broth with novobiocin (MKTT broth). After inoculation, the RVS broth was incubated at 41.5 ± 1 °C for 24 ± 3 h, and the MKTT broth at 37 ± 1 °C for 24 ± 3 h [16].

### 2.6. Plating, Identification, and Biochemical Tests

The culture obtained from selective enrichment was streaked onto two selective media: Xylose Lysine Deoxycholate Agar (XLD Agar, Kasvi, Conda S.A, Madrid, Spain) and Brilliant Green Agar (BGA, Kasvi, Conda S.A, Madrid, Spain). The XLD Agar was incubated at 37 ± 1 °C and examined after 24 ± 3 h. The BGA was incubated at 42 °C for 24 h. After this period, three characteristic colonies of BGA and XLD of each sample were streaked onto nutrient agar tubes to obtain abundant growth of the strain for further identification with biochemical tests. The biochemical tests TSI (Triple Sugar Iron Agar, Kasvi, Conda S.A, Madrid, Spain) and LIA (Lysine Iron Agar, Kasvi, Conda S.A, Madrid, Spain) were performed by inoculating pure colonies taken from nutrient agar tubes into the respective media, followed by incubation at 37 °C for 18 to 24 h. The TSI test evaluated the fermentation of sugars (glucose, lactose, and/or sucrose), gas production, and hydrogen sulphide (H_2_S) formation, based on colour changes and the presence of black precipitate. The LIA test assessed lysine decarboxylation or deamination and H_2_S production, with results interpreted according to medium colour changes and the presence of precipitate [16].

### 2.7. DNA Extraction and Analysis for the Confirmation of Salmonella spp.

Colonies testing positive for *Salmonella* spp. through biochemical test readings underwent an additional confirmation step using Polymerase Chain Reaction (PCR). For this purpose, DNA extraction was performed using thermal lysis. Isolated bacterial colonies were reactivated in BHI (Brain Heart Infusion, Conda S.A, Madrid, Spain) broth, and then 1 mL of the suspension was centrifuged at 10,000 rpm for 5 min. The supernatant was discarded, and this process was repeated until the complete formation of the bacterial pellet. The resulting pellets were resuspended in 300 µL of sterile ultrapure water and incubated in a heat block at 100 °C for 10 min. Subsequently, the tubes were chilled on ice for 10 min and centrifuged again at 10,000 rpm for 5 min. The supernatant containing the DNA was transferred to sterile tubes and used in PCRs. The primers used contained the *hilA* gene, with the following sequences; forward: CTGCCGCAGTGTTAAGGATA and reverse: CTGTCGCCTTAATCGCATGT. The equipment used was a thermocycler from Applied Biosystems by Life Technologies, model 9902 (Singapore). The cycling conditions followed the protocol described by Guo et al. (2000) [17]: 94 °C for 5 min, followed by 35 cycles of 94 °C for 1 min, annealing at 58 °C for 1 min, extension at 72 °C for 1 min, and a final extension at 72 °C for 10 min.

### 2.8. Agarose Gel Electrophoresis

A 1.5% agarose gel was prepared using TEB buffer. To allow the visualization of DNA fragments, 1 µL of ethidium bromide was added directly to the gel. In the wells designated for the marker, 2 µL of Gel Blue Juice 10X and 5 µL of 100 bp DNA Ladder were loaded. For the negative control and the samples, 2 µL of Gel Blue Juice 10× and 5 µL of the amplicon were used. All reagents came from Invitrogen and Thermo Fisher Scientific (Invitrogen, Waltham, MA, USA). Electrophoresis was performed in a chamber from Loccus Biotecnologia at 100 V, 150 mA, and 20 W for 70 min. DNA fragment visualization was carried out using a transilluminator from DNR Bio Imaging Systems (Jerusalem, Israel), model MiniBIS Pro.

### 2.9. Data Analysis

Fisher’s Exact Test was applied to assess the association between fish washing treatments (Treatment 1, Treatment 2, and Treatment 3) and the occurrence of *Salmonella* spp. contamination (presence/absence), considering a significance level of 5% and 95% confidence intervals (CI). This test is suitable for small sample sizes and was applied because of the low frequencies observed in the results. Additionally, the odds ratio (OR) was calculated to quantify the strength of association, representing the likelihood of increased or decreased contamination in one treatment compared to another. All analyses were conducted using R^®^ software version 4.2.3 (R Core Team, Vienna, Austria, 2023), with the “stats” package for Fisher’s Exact Test and the “epiR” package (Stevenson & Sergeant, 2024) [18] for OR and CI calculations.

## 3. Results

**Figure 4:**
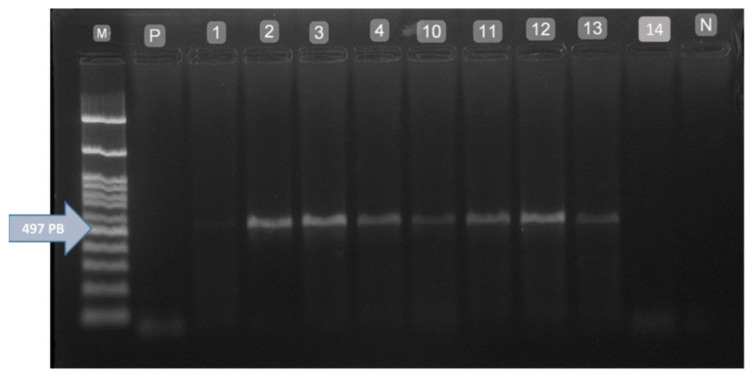
PCR products of the hyla gene (497 bp) in *Salmonella* spp. visualized on a 1.5% agarose gel. Captions: M: 100 bp marker; P: positive control using *Salmonella typhimurium* reference strain (ATCC 10,749); 1 and 14: negative samples; 2, 3, 4, 10, 11, 12, and 13: positive samples; N: negative control.

The PCR results were confirmed through the analysis of the profiles obtained in agarose gel electrophoresis, where the positive samples showed bands approximately 497 base pairs (bp) in size, consistent with the expected size for the target gene *hilA* of *Salmonella* spp. These results can be visualized in Figure 4, corresponding to the image of the agarose gel at a 1.5% concentration.

**Scheme 1:**
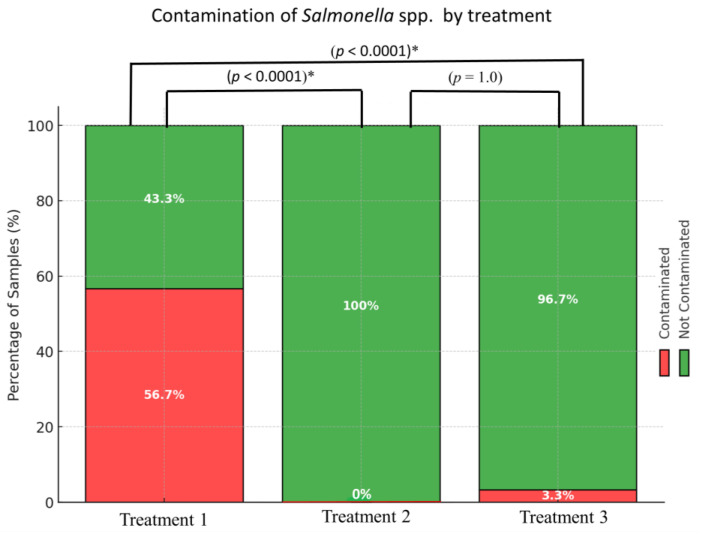
Distribution of contaminated and uncontaminated samples by treatment. The graph illustrates the absolute frequencies of contaminated and uncontaminated fish samples for each treatment. Treatment 1: Routine processing performed by the fish processing plant. Treatment 2: Enhanced processing flowchart. Treatment 3: Integration of the enhanced flowchart with the physical structure and equipment of the processing plant. Significant differences were observed between treatments 1 and 2 (*p* < 0.001), and between treatments 1 and 3 (*p* < 0.001). There was no significant difference between treatments 2 and 3 (*p* = 1). * Indicates statistical difference between treatments.

According to the confirmation obtained through the analysis of all samples subjected to gel electrophoresis, the results showed marked differences in the contamination rates by *Salmonella* spp. among the flowcharts evaluated. In treatment 1 (conventional flowchart), 56.7% of the fish samples analyzed showed contamination by *Salmonella* spp. after processing. In treatment 2 (readjusted flowchart), no samples showed contamination, indicating the absence of *Salmonella* spp. at the end of this flowchart. In treatment 3, which integrated the flowchart readjusted to the specific conditions of the meatpacking plant, a low contamination rate was indicated, with only 3.3% of the samples detected with *Salmonella* spp., as observed in Scheme 1, below.

For statistical analyses, Fisher’s exact test was performed due to the low frequency of contamination observed in the treatments, ensuring the robustness of the analyses. It was observed that the readjusted flowchart (treatment 2) significantly reduced the presence of *Salmonella* spp. compared to the conventional flowchart (treatment 1) used in fish processing plants. No samples were detected with contamination in treatment 2, reinforcing the effectiveness of the improvements made to this flowchart that resulted in the complete absence of *Salmonella* spp. contamination in the fish samples analyzed (OR = ∞, CI = [7.655, ∞], *p* < 0.001).

The significant association with the reduction in *Salmonella* spp. contamination in fish samples was also observed in the flowsheet readjusted to the specific plant conditions (treatment 3) compared to the conventional flowsheet (treatment 1) (*p* < 0.001). The confidence interval (CI = [4.67, 1622.16]) indicates a strong reduction in the probability of contamination in treatment 3 compared to treatment 1, with an estimated odds ratio (OR) of 35.62, as seen in Table 1, below.

Despite this amplitude caused by a single sample detected with *Salmonella* spp., the result reinforces the effectiveness of implementing the readjusted flowchart in the processing plant to reduce contamination. This finding highlights the potential of treatment 3 for practical application in meatpacking plants, but also highlights the need for additional validation in different industrial fish processing conditions. When comparing the readjusted flowchart (treatment 2) and the flowchart readjusted to the specific conditions of the plant (treatment 3), there are indications that the risk of contamination is practically the same, suggesting that treatment 3 maintained levels of reduction in the presence of *Salmonella* spp. similar to treatment 2 (*p* = 1.0), with minimal contamination detected. These findings suggest that the readjusted flowchart (treatment 2) not only works consistently in reducing contamination by *Salmonella* spp. during fish processing in the meatpacking plant, but also presents high efficacy when adapted to the operational conditions of the industrial plant (treatment 3).

## 4. Discussion

Fish and seafood products have been associated with foodborne outbreaks, constituting about 6 to 8% of the total confirmed foodborne disease outbreaks in recent years [19]. This incidence is considered higher when compared to the incidence of foodborne outbreaks associated with poultry and beef, which contributed an average of 3.6% and 1.9% of the total outbreaks from 2011 to 2017 [19]. Based on the main pathogens associated with fish products, *Salmonella* has been the leading bacterial etiology for outbreaks linked to fish, highlighting significant challenges for food safety and the need for better control strategies in the seafood supply chain [20].

*Salmonella* spp. has been a frequently detected pathogen in fish products in several countries. In Brazil, according to a study by Fernandes et al. [7], *Salmonella* was detected in all areas of the fish processing environment. In the study by Cunha Neto et al. [12], *Salmonella* spp. was found in frozen fish belly flaps exposed for commercialization. In addition to Brazil, other countries also report this contamination, such as the study by Pal et al. [21], which analyzed frozen catfish fillets purchased from retail stores in Vietnam and found *Salmonella* in 42% of the samples. Another study conducted in the United States in fish processing plants examined the microbiological quality of frozen catfish fillets and detected *Salmonella* in 5.3% of the samples [22].

There are several factors that contribute to the persistence of *Salmonella* spp. on the surface of fish after processing. The receiving stage is one of the critical points in industrial processing [7], since fish often arrive at the plant already contaminated externally, such as in their scales, in addition to being loaded with organic matter, including mud, soil, and mucus [23]. The effectiveness of sodium hypochlorite (NaClO) during washing with 5 ppm chlorinated water is significantly reduced due to the high load of organic matter present on the fish. This organic matter reacts with the available NaClO, decreasing its effective concentration. This effect was reported by Narayan et al. [24], who observed a reduction of approximately ten times in the concentration of free chlorine in the presence of 5% organic matter. Reis et al. [8] also demonstrated that the addition of 1% to 5% organic matter in a solution containing 5 ppm chlorine reduced the availability of free chlorine to only 1.43 ppm. This reinforces the need to perform a preliminary wash to remove dirt (organic matter) at the receiving stage, before washing with 5 ppm chlorinated water, which can significantly reduce the risk of cross-contamination associated with the entry of fish into the clean processing area still with scales, potentially carrying pathogens such as *Salmonella*. Our research identified that the chlorinated water baths used in this industry have an extremely short duration of available active chlorine, typically only a few seconds, which may be insufficient to inactivate *Salmonella* spp. The exposure time and the concentration of sodium hypochlorite are important factors for the effective inactivation of and/or reduction in *Salmonella* spp. [9]. According to Reis et al. [8], a significant reduction in *Salmonella* spp., greater than 5 logs in the presence of organic matter, requires the sodium hypochlorite to remain in contact with the pathogen for approximately 30 min.

Gills and scales are recognized as potential sources of contamination in fish [7,25], and when handled in the clean area, they can cause cross-contamination, compromising the microbiological quality of the final product [5]. Therefore, it can be suggested that the removal of gills and scales carried out outside the clean area, as in procedures implemented in the enhanced processing of fish (treatments 2 and 3, as seen in Figure 1 and Figure 2), was responsible for the success in eliminating and/or reducing the presence of *Salmonella* spp. in the final product.

Despite the common practice of washing fish with 5 ppm chlorinated water in processing facilities (treatment 1), many fish still enter the clean area with scales, which can serve as carriers of microorganisms and contribute to cross-contamination [7,25,26]. Studies have shown that inadequate handling practices and equipment sanitation, particularly in the evisceration area, are critical factors influencing the microbiological safety of the final product [5]. In one case, up to 50% of raw processing samples and over 15% of finished products were contaminated with *Salmonella* spp., highlighting persistent failures in sanitary control procedures [27].

The results of the analyses implemented on samples subjected to conventional processing (treatment 1, Figure 1) show that there is contamination by *Salmonella* spp. in 56.7% of the samples analyzed in this group. These results were found even after the use of 5 ppm chlorinated water. However, factors such as the very quick removal of the viscera and the fact that most fish had ruptured viscera during this stage were observed, which may have favoured cross-contamination, as this organ can harbour a higher concentration of the pathogen [28]. These actions compromised more than half of the samples with *Salmonella* spp. Additionally, another study [29] investigated the occurrence of *Salmonella* spp. in tilapia processing plants intended for the production of ready-to-eat sashimi, demonstrating the presence of the pathogen in different areas of the production line, including the descaling and evisceration steps. Exactly the same critical points were identified in the present study as the main factors contributing to cross-contamination and, consequently, to the contamination of the final product. The isolation rates of *Salmonella* spp. in these steps ranged from 10.2% to 36.1%. Moreover, the pathogen was also identified on processing surfaces, with an isolation rate of 10.5%, reinforcing the importance of strict sanitary control throughout the production chain. Finally, *Salmonella* spp. was also detected in the final products, with isolation rates ranging from 4.6% to 36.1%, highlighting the risk of contamination persisting throughout processing and reaching the end consumer. These findings reinforce the need for adjustments in processing protocols to mitigate microbiological contamination risks. In Brazil, this concern is even more relevant considering the current legislation, Normative Instruction No. 161, dated 1 July 2022, which establishes the mandatory absence of *Salmonella* in fish and their derivatives, including seafood [30].

An intervention was implemented (treatment 2, Figure 2), where the viscera were carefully removed to prevent rupturing, ensuring that intestinal contents did not come into contact with the fish carcasses. However, if the viscera were ruptured, the handlers were trained to discard the fish outside the processing area, and immediately clean all surfaces in contact with the fish (countertops, cutting boards) in the evisceration area with 25 ppm chlorinated water [14]. Knives and sharpening steels were replaced, and handlers’ gloves were changed. This ensured the success of treatment 2 (enhanced, Scheme 1) to prevent cross-contamination during processing, which resulted in 100% of the samples being free from *Salmonella* spp. contamination.

The success achieved with treatment 2 (enhanced) was also observed in treatment 3, which used the enhanced flowchart procedure, executed with the equipment available in the industry. A low contamination rate of only 3.3% of the samples was observed, compared to the 56.7% observed in treatment 1, as seen in Table 1 These results indicate that, even with a contamination rate of 3.3%, which is considered very low, there was no statistically significant difference. Therefore, the main factor to be considered for the success of the process was the modification of the flowchart, with the separation of processing stages into dirty areas (gill and scale removal) and clean areas (evisceration and filleting). Thus, the implementation of the enhanced flowchart can be a viable and efficient strategy to improve contamination control in the fish slaughter industry, without the need for large investments in new equipment. This feasibility is also evidenced by the analysis of operational costs, as there is no significant increase in total costs with the adoption of treatments 2 and 3 (Figure 2 and Figure 3) compared to the conventional treatment (Figure 1). The main difference between the methods lies in the use of a high-pressure water pump (treatment 2, Figure 2), a low-cost piece of equipment that is generally already available in most slaughterhouses, and in the need for greater attention during the evisceration step (treatments 2 and 3, Figure 2 and Figure 3), especially to avoid rupturing the viscera. This extra care makes the process slower, which justifies the need for more workers to maintain production line efficiency. At the slaughterhouse in which the study was conducted, evisceration is normally carried out by two workers; due to the slower process caused by this additional care, four workers were assigned to perform this task in treatments 2 and 3. However, over time, workers tend to develop greater skill in carrying out this task, which can help optimize the process.

Moreover, as workers develop greater skill over time, the process becomes not only more efficient but also more consistent, which contributes to improved food safety outcomes. This, in turn, can significantly reduce contamination rates [27], reinforcing the potential of the process to meet more stringent microbiological control standards. This improves the ability of the domestic industry to meet the requirements of international markets, expanding export opportunities and strengthening its position in the global market.

## 5. Conclusions

The results of this study demonstrate that the proper separation of processing areas between dirty zones (gill and scale removal) and clean zones (evisceration and filleting), combined with the training of the evisceration team, is essential for controlling *Salmonella* spp. contamination in native round fish. The modified processing flowchart proved effective even without equipment replacement, as long as the processing steps were properly separated. Additionally, the use of 5 ppm of free residual chlorine was effective under these conditions. It is recommended that fish processing plants reorganize their processing line layout to physically separate dirty and clean steps, implementing structural barriers and promoting continuous staff training as key control strategies.

## Figures and Tables

**Table 1:** Comparison between treatment groups (T1, T2, T3) based on the number and percentage of positive and negative cases. Odds ratios (OR) with 95% confidence intervals (CI) and *p*-values are presented for each pairwise comparison.

Treatments Compared	Samples		*OR* (*IC*)	*p*-Value
	Positives (n, %)	Negatives (n, %)		
T1 vs. T2	17 (56.7%) vs. 0 (0%)	13 (43.3%) vs. 30 (100%)	* ∞, (7.655, ∞)	6.19 × 10^7^
T1 vs. T3	17 (56.7%) vs. 1 (3.3%)	13 (43.3%) vs. 29 (96.7%)	35.62 (4.67, 1622.16)	7.95 × 10^6^
T2 vs. T3	0 (0%) vs. 1 (3.3%)	30 (100%) vs. 29 (96.7%)	** 0 (0, 39.00)	1.0

**Notes:** * Infinite Odds Ratio reflects the complete effectiveness of treatment 2 in reducing contamination by *Salmonella* spp. in all fish samples analyzed, compared to treatment 1. This reduction highlights the potential of the readjusted flowchart to contribute to microbiological safety; therefore, readjustments are recommended to be tested in different cold storage plants. ** Odds Ratio zero reflects the absence of contaminated samples in treatment 2 compared to treatment 3. Despite a single contaminated sample in treatment 3, the adaptation of the readjusted flowchart maintained an effective reduction in contamination, demonstrating operational viability even in industrial plant conditions.

## Data Availability

The original contributions presented in this study are included in the article, and additional questions can be directed to the corresponding author.
